# Video-based classroom insights: Promoting self-regulated learning in the context of teachers’ professional competences and students’ skills in self-regulated learning

**DOI:** 10.1007/s42010-023-00189-8

**Published:** 2023-12-19

**Authors:** Amina Rosenthal, Carmen Nadja Hirt, Tabea Daria Eberli, Johannes Jud, Yves Karlen

**Affiliations:** Institute of Education, University of Zurich, Kantonsschulstrasse 3, 8001 Zurich, Switzerland

**Keywords:** Self-regulated learning, Metacognition, Strategy instruction, Classroom observation, Teachers’ knowledge, Teachers’ beliefs, Selbstreguliertes Lernen, Metakognition, Strategievermittlung, Unterrichtsbeobachtung, Wissen der Lehrkräfte, Überzeugungen der Lehrkräfte

## Abstract

Despite the significance of self-regulated learning as an important educational goal, teachers face difficulties in fostering students’ skills in self-regulated learning (SRL). Teachers exhibit variability in their capacity to foster SRL. There is no guarantee that students consistently benefit from their teachers’ promotion of SRL. This study aims to address this issue by examining (1) how teachers promote SRL, (2) the relationship between teachers’ professional competences and their promotion of SRL, and (3) the association between teachers’ promotion of SRL and students’ SRL. Data from *N* = 54 teachers and their *N* = 823 lower secondary school students were analysed using online questionnaires, knowledge tests, and video recordings. The analysed video data reveals that teachers foster SRL predominantly implicitly, invest most of the time in promoting metacognitive strategies and primarily design learning environments that foster student support. Overall, only a few significant correlations were found between teachers’ professional competences and their promotion of SRL. Further, the results indicate no clear correlation pattern between teachers’ promotion and students’ skills in SRL. Further research should shed more light on the relationship between teachers’ promotion of SRL and students’ SRL to better understand whether and how they might be related.

## Abstract (de)

Trotz der Bedeutung des selbstregulierten Lernens (SRL) als wichtiges Bildungsziel, haben Lehrkräfte Schwierigkeiten, die Fähigkeiten der Schüler:innen zum SRL zu fördern. Lehrkräfte zeigen unterschiedliche Fähigkeiten, selbstreguliertes Lernen zu fördern. Es ist nicht garantiert, dass Schüler:innen von der Förderung des selbstregulierten Lernens durch ihre Lehrkräfte profitieren. Daher untersucht diese Studie (1) wie Lehrkräfte SRL fördern, (2) die Beziehung zwischen den professionellen Kompetenzen der Lehrkräfte und ihrer Förderung des SRL und (3) den Zusammenhang zwischen der Förderung des SRL durch die Lehrkräfte und dem SRL der Schüler:innen. Die Daten von *N* = 54 Lehrkräften und ihren *N* = 823 Schüler:innen der Sekundarstufe I wurden anhand von Online-Fragebögen, Wissenstests und Videoaufnahmen analysiert. Die analysierten Videodaten zeigen, dass die Lehrkräfte das SRL überwiegend implizit fördern, die meiste Zeit in die Förderung metakognitiver Strategien investieren und primär Lernumgebungen gestalten, die die Schüler:innen unterstützen. Insgesamt wurden nur wenige signifikante Korrelationen zwischen den professionellen Kompetenzen der Lehrkräfte und ihrer Förderung des SRL gefunden. Darüber hinaus zeigen die Ergebnisse kein klares Korrelationsmuster zwischen der Förderung der Lehrkräfte und dem SRL der Schüler:innen. Weitere Untersuchungen sollten die Beziehung zwischen der Förderung des SRL durch die Lehrkräfte und dem SRL der Schüler:innen genauer beleuchten, um besser zu verstehen ob und wie diese zusammenhängen könnten.

## Introduction

Self-regulated learning (SRL) contributes to academic and professional success, and it extends beyond these domains (Dent and Koenka 2016). SRL encompasses cognitive, metacognitive, and motivational-emotional skills students need to gain knowledge and overcome learning challenges (Boekaerts 1999; Pintrich 2000; Zimmerman 2000). SRL is a complex, effortful process that requires acquiring, coordinating, and consolidating metacognitive knowledge and different strategies (Pressley et al. 1987). Teachers are essential in developing students’ SRL skills (Karlen et al. 2020). They can promote SRL directly (e.g., though explicit or implicit explanations of strategies) and indirectly (by creating an environment that promotes SRL) (Dignath and Veenman 2021). Video studies reveal heterogeneous promotion of SRL by teachers, with a primary focus on cognitive strategies and infrequent explicit promotion of SRL (e.g., Dignath and Büttner 2018; Kistner et al. 2010; Spruce and Bol 2015). Teachers’ professional competences in SRL among teachers have been identified as explanatory factors for the variations in their promotion of SRL (e.g., Gordon et al. 2007; Wilson and Bai 2010). However, teacher competence aspects (e.g., knowledge beliefs, motivation) in SRL showed inconsistent relationships to teachers’ self-reported and observed SRL promotion (Karlen et al. 2020; Kramarski and Heaysman 2021; Spruce and Bol 2015). Additionally, the relationship between teachers’ SRL promotion and students’ SRL skills yielded mixed findings, with some studies showing positive (e.g., Kistner et al. 2010; Zepeda et al. 2019) and others showing no or even negative relations (e.g., Depaepe et al. 2010; Dignath-van Ewijk et al. 2013). These inconsistent results necessitate further investigation. This study addresses these objectives by examining the frequency and variety of teachers’ observed SRL promotion, as well as the links between teachers’ professional competences in SRL and their promotion, and between teachers’ SRL promotion and students’ SRL skills.

## Theoretical background

### Self-regulated learning

In recent decades, different SRL definitions and models have been presented. Researchers agreed that self-regulated learners actively control their cognition, motivation, emotions, and behaviour to achieve their goals (Boekaerts 1999; Zimmerman 2000). Self-regulated learners believe that SRL skills are malleable and recognise the crucial role of deliberately employing strategies to achieve high academic success (Hertel and Karlen 2021). The models by Boekaerts (1999) and Pintrich (2000) highlight the various types of metacognitive, cognitive, resources, motivational, and emotional strategies that are important for self-regulated leaners. There is consensus that self-regulated learners are required to acquire, coordinate, and apply different strategies to enhance their learning (Pressley et al. 1987). To effectively apply and combine strategies, self-regulated learners need metacognitive knowledge (Boekaerts 1999; Pressley et al. 1987). Metacognitive knowledge (MK) encompasses the comprehension of diverse strategies, knowing when and how to apply them to different tasks, and evaluating their effectiveness in comparison to alternative strategies. MK empowers self-regulated learners to make informed decisions regarding their strategies (Pintrich 2002). It allows learners to inherently connect strategies with the alignment of the task (Pressley et al. 1987). Overall, SRL is an effortful process that places high demands on learners and results in a high cognitive load, particularly for learners with little SRL experience (Schuster et al. 2020). For this reason, teachers must offer guidance and support to learners throughout the SRL process (Karlen et al. 2020).

### Promotion of self-regulated learning

Teachers’ instructional practices can significantly influence the development of students’ SRL skills (Karlen et al. 2020). Teachers can promote SRL by providing direct (explicit, implicit) and indirect instructions. Explicit direct instruction involves teachers clearly explaining and demonstrating strategies and providing MK about strategies. Teachers’ implicit direct instructions induce students to apply a strategy without any information about its use or benefit. These instructions may not necessarily impact students’ SRL, as students first need to acquire MK about strategies before recognising the benefits of strategies themselves (Dignath and Veenman 2021). Indirect SRL promotion includes designing powerful learning environments for practising strategies (Dignath and Veenman 2021) and is based on constructivist views on learning and is student-directed (De Corte et al. 2004; Pintrich 2000). Such learning environments enable student autonomy in SRL (self-determination), support learning through real-life contexts (value), encourage SRL engagement (success expectation), promote active collaboration (cooperative learning), support positive emotions and relationships (student support), and activate prior knowledge (constructivist learning) (Dignath et al. 2022; Hugener et al. 2006).

Video-based studies on teachers’ SRL promotion are rare, especially those that systematically distinguish between direct and indirect promotion. Mainly, these studies examine teachers from one school subject and revealed that teachers promote SRL implicitly rather than explicitly (e.g., Dignath and Büttner 2018; Kistner et al. 2010; Spruce and Bol 2015). Furthermore, teachers promote predominantly cognitive than other strategies and foster SRL limited indirect. When teachers provided indirect promotion, they created more conducive learning environments for students’ constructive learning than other approaches (Dignath and Büttner 2018; Kistner et al. 2010). Most results show that teachers differ in their SRL promotion (Spruce and Bol 2015). Teachers’ professional competences might explain these variations.

### Teachers’ professional competences and the promoting of self-regulated learning

Several models about teachers’ professional competences in SRL have been presented and include teachers’ knowledge, beliefs, and motivational aspects as crucial parts of their dual role as learners and agents of SRL (e.g., Karlen et al. 2020; Kramarski and Heaysman 2021). The following sections describe teachers’ professional competences as self-regulated learners and their professional knowledge and beliefs of SRL.

#### Teachers as self-regulated learners

Teachers as self-regulated learners possess an awareness of their strengths and weaknesses in SRL and a deep understanding of the challenges associated with SRL (Gordon et al. 2007). In the classroom, teachers’ experiences as learners of SRL might enable them to act as metacognitive role models for their students and, thereby, effectively demonstrate and explain strategies (Karlen et al. 2020; Pintrich 2002). Further, one can assume that teachers with metacognitive awareness and self-regulation competences might gain a deeper insight into their students’ SRL processes. This insight equips them to proficiently recognise and tackle their students’ requirements and difficulties with SRL (Karlen et al. 2020, 2023; Paris and Winograd 2003). Only a few studies have empirically explored the relationship between teachers’ competences as self-regulated learners and their SRL promotion. Those studies found positive correlations between teachers’ own SRL competences and their self-reported design of learning environments (Gordon et al. 2007) as well as their knowledge of how to teach metacognitive strategies (Wilson and Bai 2010), and indirect effects to their self-reported SRL promotion indirectly via self-efficacy (Karlen et al. 2023).

#### Teachers’ knowledge about self-regulated learning

Teachers’ professional knowledge is often divided into content knowledge (CK) about the teaching content and pedagogical content knowledge (PCK) about how to teach content to students (Shulman 1987). Applied to the context of SRL, with SRL defined as “content”, CK-SRL encompasses teachers’ knowledge and understanding of the concept of SRL. It includes familiarity with terminology, theoretical models, strategies, and the different components of SRL, such as metacognition. PCK about SRL (PCK-SRL) encompasses teachers’ knowledge of the various ways to promote SRL (Karlen et al. 2020). The findings on the relationship between teachers’ knowledge and their SRL promotion are inconsistent (e.g., Lawson et al. 2019). Some researchers have reported significant connections between teachers’ knowledge and their SRL promotion (e.g., Barr and Askell-Williams 2019; Karlen et al. 2020), whereas others have reported no relation between teachers’ knowledge and their observed SRL promotion (Dignath-van Ewijk 2016; Spruce and Bol 2015). The distinction between CK-SRL and PCK-SRL may be relevant, as the correlation between PCK-SRL and teacher action seems to be stronger than between CK-SRL and teachers’ SRL promotion (Barr and Askell-Williams 2019; Karlen et al. 2020).

#### Teachers’ beliefs about self-regulated learning

Beliefs create a cognitive framework that guides learners in interpreting their experiences. They control how learners perceive their knowledge, abilities and what predictions (e.g., expectations of success) they make (Lawson et al. 2019). Beliefs contribute to the value learners ascribe to learning and influence motivation and learning behaviour (Fives and Buehl 2012). Important beliefs are teachers’ malleability and relevance mindsets about SRL (Hertel and Karlen 2021). Malleability mindsets about SRL are core assumptions about SRL abilities that range from fixed to growth mindsets. Individuals with a fixed mindset believe that SRL skills are innate and unchangeable. In contrast, individuals with a growth mindset believe that SRL skills can be acquired and improved through effort and practice. Relevance mindsets about SRL span a continuum from less to more relevant, indicating the extent to which individuals recognise and value the importance of SRL for their academic development (Hertel and Karlen 2021). Mindsets about SRL are attached to (pre-service) teachers’ strategy use and knowledge about SRL (Hertel and Karlen 2021) and indirectly to their self-reported SRL promotion via intrinsic value (Karlen et al. 2023). Studies on the relationship between teachers’ general beliefs (about SRL) and their SRL promotion present an inconsistent picture. Researchers reported positive connections between teachers’ beliefs and teachers’ self-reported SRL promotion (Dignath-van Ewijk 2016; Vosniadou et al. 2021) as well as no relation with their videotaped SRL promotion (Dignath and Büttner 2018; Spruce and Bol 2015).

### Teachers’ promotion of self-regulated learning and students’ self-regulated learning

Studies exploring the relationship between teachers’ SRL promotion and students’ SRL skills yielded mixed results. Some studies reported positive relationships (e.g., Depaepe et al. 2010; Moely et al. 1992), while others found no or negative relationships (e.g., Hamman et al. 2000; Heirweg et al. 2021; Karlen 2016). The diverse outcomes of promoting SRL can be attributed to several factors. These include the use of different measurement tools to assess SRL promotion and students’ SRL outcome, variations in the duration of teachers’ SRL promotion, variations in student-perceived SRL promotion, contextual factors like school vision that might influence the effect of teachers’ SRL promotion, and the lack of a systematic distinction between direct and indirect promotion. Comparing these results and drawing clear conclusions is challenging. Further research is needed to understand this complex relationship.

## Aim of the study

Examining how teachers promote SRL in their classrooms, which has led to mixed results, largely depends on self-report questionnaires (Karlen et al. 2020; Kramarski and Heaysman 2021; Spruce and Bol 2015). A few of studies have observed teachers’ SRL promotion using methods such as video recording (e.g., Dignath and Büttner 2018; Kistner et al. 2010). Our video study aims to replicate and expand upon prior research by examining various teachers’ professional competences as potential predictors of SRL promotion. Additionally, we explore the relationship between teachers’ SRL promotion and students’ SRL by answering the following questions:

1. To what extent do teachers promote SRL in classroom?
2. How are teachers’ professional competences related to their SRL promotion in classroom?
3. How are teachers’ direct and indirect SRL promotion related to students’ SRL?

Previous research on teachers’ video-taped promotion of SRL revealed that teachers predominantly foster strategies implicitly, primarily focusing on promoting cognitive strategies (Dignath and Büttner 2018; Kistner et al. 2010; Spruce and Bol 2015). As a result, we assume that in this study, (H1a) teachers primarily promote strategies implicitly and (H1b) predominantly emphasise cognitive strategies in their classroom.

The empirical findings concerning the relationship between teachers’ professional competences in SRL and their SRL promotion are inconsistent (Barr and Askell-Williams 2019; Gordon et al. 2007; Wilson and Bai 2010). Despite these discrepancies, we postulate, based on theoretical models of teachers’ professional competences in SRL, particularly as suggested by Karlen et al. (2020), that teachers’ professional competences in SRL positively correlate with their SRL promotion (H2).

Research examining the connection between teachers’ promotion of SRL and students’ SRL skills has produced inconsistent findings, ranging from positive to negative correlations (Dignath and Veenman 2021; Heirweg et al. 2021; Karlen 2016). Consequently, no clear hypotheses are formulable, and this question is explored exploratorily.

## Method

### Participants and procedure

This study is embedded in a longitudinal intervention study on teachers’ professional competences in SRL with a quasi-experimental pre-, post-, and follow-up design. This study presents the data from the first measurement point. Participants were lower secondary teachers (*N* = 54; 61% female) and their students (*N* = 823; 45% female; age: *M* = 13.68, *SD* = 0.86) from 35 different schools in German-speaking Switzerland. Teachers were on average *M* = 34.56 years old (*SD* = 8.95, min = 19, max = 51) and had an average professional experience of *M* = 16.48 years (*SD* = 10.13, min = 1, max = 36). An average of *M* = 17 students (*SD* = 5.16, min = 3, max = 25) per class participated in the study. Teachers’ lessons taught different school subjects (native language [German], foreign languages [French and English], mathematics, biology, geography, and history-religion) were videotaped before the intervention.

Lower secondary school principals from [Germen-Speaking Switzerland] were contacted via e‑mail, and a call for participation was launched via educational authorities in different provinces. Participation was voluntary, and teachers and students were informed about the data collection procedures. Students’ parents had to consent, and all procedures followed the ethical guidelines of the Swiss National Science Foundation. The Ethics Committee of the University of Applied Sciences and Arts Northwestern Switzerland and several educational authorities from different provinces have approved this study. After receiving informed consent, teachers and students completed online questionnaires before the intervention. The students did so during the lessons with the teachers’ administrative support.

### Measures

Using a multimethod approach, data were collected through online questionnaires, knowledge tests, and video recordings. Descriptive statistics and internal consistencies of the SRL scales for both teachers and students are presented in Table 1.

**Table 1:** Descriptive statistics and internal consistencies

Construct	No. of Items	α	*n*	*M*	*SD*	Skew	Kurtosis	Observed range	Possible range
*Teachers’ SRL competences*									
Metacognitive awareness	4	0.84	54	4.57	0.74	−0.34	−0.08	2.5–6.0	1.0–6.0
Metacognitive regulation skills	5	0.80	54	4.62	0.39	−0.55	0.16	3.0–5.6	1.0–6.0
Motivational regulation skills	4	0.87	54	4.59	0.78	−0.01	−0.53	2.8–6.0	1.0–6.0
CK-SRL	19	–	54	4.50	1.23	−0.24	−0.42	2.0–7.0	0.0–10.0
CK-SRL Meta^a^	1	–	54	2.33	1.12	0.73	−0.18	1.0–5.0	0.0–8.0
PCK-SRL	32	0.91	54	0.77	0.20	−1.93	3.60	0.1–1.0	0.0–1.0
Malleable mindsets	3	0.68	54	5.41	0.56	−0.64	−0.64	4.0–6.0	1.0–6.0
Relevance mindsets	3	0.78	54	4.62	0.86	−0.66	0.44	2.0–6.0	1.0–6.0
*Students’ SRL skills*^*b*^									
MK-Meta	24	0.80^c^	51	0.65	0.08	−0.68	0.25	0.4–0.8	0.0–1.0
Cognitive regulation skills	4	0.77^c^	51	2.98	0.15	−0.26	−0.25	2.6–3.3	1.0–4.0
Motivational regulation skills	4	0.79^c^	51	2.61	0.17	0.16	0.12	2.2–3.0	1.0–4.0
Strategy use	7	0.69^c^	51	2.96	0.16	−0.07	−0.64	2.6–3.3	1.0–4.0

*α* Cronbach’s alpha coefficients, *n* number of cases, *M* mean, *SD* standard deviation

^a^ ICC = 0.84 (Cohen’s Kappa (*k*))

^b^ Aggregated data

^c^ Cronbach’s alpha coefficients on student level

#### Observation of the promotion of self-regulated learning

Each teacher was videotaped for one lesson to investigate teachers’ SRL promotion. Teachers were asked to “Show a ‘typical, everyday lesson’ that exemplifies how you promote interdisciplinary competences (e.g., SRL) in your lessons. It should be an introductory lesson (not a practice lesson).” After the lesson recording, teachers were questioned to rate (on a scale from 1 = does not apply at all to 6 = fully applies) how representative the lesson was of their regular teaching style. The teachers’ responses had an average *M* = 4.69 (*SD* = 0.81, min. = 3.00, max. = 6.00). The videos were standardised to 45-minute school lessons. Teachers’ direct and indirect SRL promotion was coded following the instrument “*A*ssessing How *T*eachers *E*nhance *S*elf-regulated Learning” (ATES; Dignath et al. 2022). Low-inference coding measured the quantity of explicit and implicit promotion of strategies (number and time invested in promoting different types of strategies) to rate teachers’ direct SRL promotion. Teachers’ verbal instructions and non-verbal behaviours to promote SRL directly were coded during video analysis—high-inference coding rated teachers’ indirect SRL promotion (learning environment characteristics). In contrast to low-inference coding, high-inference coding requires increased interpretation by raters in the coding process to qualitatively assess certain observed events. Therefore, high-inference coding teachers’ indirect SRL promotion was conducted at the end of each video (Dignath-van Ewijk et al. 2013; Kistner et al. 2010).

For coding *teachers’ direct SRL promotion*, serial numbers for each initialised strategy document teachers’ promotion of one of the five strategy types: cognition, metacognition, resources, motivation, and emotion regulation, which refer to literature by Boekaerts (1999) and Pintrich (2000). Teachers’ promoted strategy type was differentiated into sub-type strategies (e.g., metacognitive strategies were differentiated into goal setting and planning, monitoring, evaluation and reflection, or regulation). Furthermore, it was determined whether the promotion was explicit or implicit. The duration of the strategy promotion was documented as less than 15 s (< 15 sec.) or as the exact period if the instruction lasted longer than 15 s. For this, two raters were trained for 63 h. Upon achieving a Cohen’s kappa value *κ* = 0.83, the raters coded the remaining videos separately.

For coding *teachers’ indirect SRL promotion,* a high-inference rating scale with six constructs and 21 items was used (see an example item for each construct in the Appendix, Table 6). Each construct consists of three or four items and was rated on a scale from 1 (low) to 4 (high). Two raters were trained for this and coded 30% of the videos to determine interrater reliability (*ICC* = 0.79). One rater coded the remaining videos.

#### Teachers’ questionnaire

*Own SRL skills* were assessed with the three SRL subdimensions: (a) metacognitive awareness, (b) metacognitive regulation skills, and (c) motivational regulation skills, introduced by Karlen et al. (2023). Schraw and Dennison’s (1994) subscale was used to assess teachers’ metacognitive awareness about the effective use of strategies (four items, e.g., “I find myself using helpful learning strategies automatically.”). Metacognitive regulation skills were assessed with a validated scale (Karlen et al. 2023) consisting of five items (e.g., “I can judge well which strategies I need to use to achieve my goals.”). Motivational regulation skills were assessed with a validated scale (Karlen et al. 2023) consisting of four items (e.g., “When my motivation wanes, I can influence it positively.”). All items were rated by teachers on a six-point scale (1 = does not apply at all; 6 = fully applies).

*CK-SRL* (knowledge and understanding of the SRL concept) was assessed through a multiple-choice (MC) knowledge test (Karlen et al. 2020). The content of the MC test includes, for example, questions about SRL, motivation, strategies, and mindsets. A total of ten questions (19 Items) could be answered using true-false or single-choice options. For each correct question, one point was scored. A sum score ranged from 0 (no or low CK-SRL) to 10 (high CK-SRL).

*CK-SRL about metacognition (CK-SRL meta) *was assessed with the open-end question, “What do you understand by metacognition?” (analogous to, e.g., Wilson and Bai 2010). The open-end question captures teachers’ knowledge and understanding of metacognition. Furthermore, the open response format provides insights into the extent to which their conceptual understanding of the metacognition process contains misconceptions. For the analysis of the teachers’ responses, a coding framework was developed: a) in a deductive way from a priori categories derived from the literature (e.g., Pintrich 2000; Zohar 2004) and b) in an inductive way through categories derived from teachers’ responses. Based on Zohar (2004), we included (a) planning, (b) goal setting, (c) monitoring, (d) evaluation, (e) reflection, and (f) regulation. Teachers received one point for each category (0–6 points). Further, the overall quality of the teachers’ answers was coded (0 = no answer or misconceptions; 1 = undifferentiated answer, which some incorrect statements; 2 = differentiated and correct answer). To receive a point, teachers had to mention or describe a metacognitive category explicitly. Points for the answer quality are based on the level of differentiation and precision with which the teachers explained metacognition. Thus, an answer that names all six categories and describes them in a differentiated and correct way scores a maximum of 8 points (0 = low CK-SRL Meta to 8 = high CK-SRL Meta). Two raters independently dual-coded teacher answers, and after achieving interrater reliability (Cohen’s kappa) of *k* = 0.89, one rater coded the remaining responses. Both raters were part of the study project and comprehensively understood metacognition. They reached a consensus on their conceptual understanding through collaborative training sessions.

*PCK-SRL* was assessed with an available knowledge test (Karlen et al. 2020). This test includes four scenarios that describe different situations addressing the implementation of SRL (introducing SRL to a class with experiences in SRL; introducing new strategies; fostering metacognitive skills; introducing a learning journal). Seven different action options were provided for each scenario, varying in degree of effectiveness. Experts’ judgments were used as an external benchmark for scoring the tests and estimating the relative relationships between the potential pairs of actions (pair comparison). A paired comparison (e.g., action A is more useful than action B in the given situation) was scored as correct if a teacher’s judgment corresponded with the experts’ ratings (1 point) and incorrect if a judgment on a paired comparison contrasted the experts’ ratings (0 points). An overall mean score was computed ranging from 0 (no pair comparisons solved correctly; low PCK-SRL) to 1 (all pair comparisons solved correctly; high PCK-SRL).

*Malleability mindsets SRL *were assessed with a validated scale developed by Hertel and Karlen (2021). The scale consists of three items that incorporate a six-fold scale (e.g., “Everyone has a certain ability to self-regulate their learning, and this … (1) cannot be changed to (6) can be changed.”). Higher values represented stronger endorsements of a growth mindset about SRL.

*Relevance mindsets SRL *were assessed with a validated scale from Hertel and Karlen (2021). The scale consists of three items that incorporate a six-fold scale (e.g., “For success in school, self-regulated learning is … (1) not a necessary prerequisite to (6) a necessary prerequisite.”). Higher values represented stronger endorsements in the relevance of mindsets about SRL for school achievement.

#### Students’ questionnaire

**Fig. 1:**
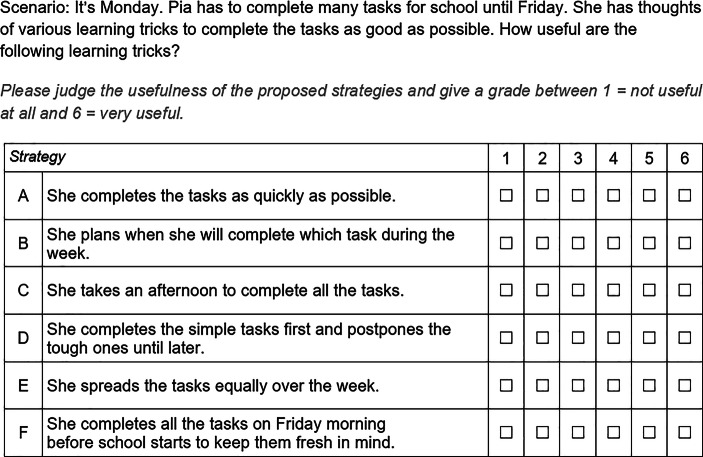
Example Task in Students’ Questionnaire: Metacognitive Knowledge (MK-Meta) about Metacognitive Strategy use. (Examples pair of strategies for this scenario that are rated by experts and used for the MK-Meta score: B > A, B > C, B > D, B > F, C > F, E > A, E > C, E > D, E > F)

*M*e*tacognitive knowledge about metacognitive strategy use (MK-Meta)* was assessed with a newly developed test based on similar tests for other MK domains (e.g., Maag Merki et al. 2013). The MK-Meta test includes four scenarios related to using metacognitive strategies: The first scenario involves planning the learning process and setting goals; the second and third scenarios involve addressing students’ ability to monitor and regulate their learning; the fourth scenario refers to evaluating the learning process. For each scenario, students had to evaluate the usefulness of six to seven different strategies for reaching the intended learning goal (see example in the Appendix, Fig. 1). Experts’ judgments were used as an external benchmark for scoring tests and building pairs between the strategies (pair comparisons; strategy B is more useful than strategy A). The MK-Meta score expresses the correspondence between the external benchmark and students’ answers. A paired comparison was scored correct if a student’s judgment corresponded with the experts’ ratings (1 point) and incorrect if the judgement contrasted the experts’ ratings (0 points). An overall mean score was computed ranging from 0 (low MK-Meta) to 1 (high MK-Meta), reflecting students’ MK about the relative strengths and limitations of metacognitive strategies for reaching specific learning goals.

*Cognitive regulation skills *were assessed with four items (e.g., “I can combine new information well with what I already know”). The scale represents students’ ability to use cognitive strategies to regulate the processing, storage, and retrieval of information. The students responded on a scale from 1 (does not apply at all) to 4 (fully applies). The scale was initially used in a prior study to assess teachers’ skills in regulating their information processing as SRL learners and has been adapted for use with students (Karlen et al. 2023).

*Motivational regulation skills* were assessed with four items (e.g., “I can start learning even when I would rather do something else”). The scale represents students’ ability to use motivational strategies to regulate their motivation. The students responded on a scale from 1 (does not apply at all) to 4 (fully applies). The scale was initially used in a prior study to assess teachers’ skills in regulating their motivation as SRL learners and has been adapted for use with students (Karlen et al. 2023).

*Self-reported strategy use *was assessed with a new scale including seven items, developed based on the theoretical and empirical literature on SRL strategies (Pintrich 2000). A short introductory text was presented: “When you think about your learning, to what extent do the following statements apply to you?” All items were rated on a scale ranging from 1 (does not apply at all) to 4 (fully applies). The scale includes cognitive (e.g., “During learning, I use learning strategies to better understand the content.”), metacognitive (e.g., “While learning, I monitor whether I am on the right track.”), and motivational strategies (e.g., “Before I start learning, I motivate myself.”).

#### Analyses

A priori power analysis in G*Power 3.1 was calculated (Faul et al. 2007). For a moderate correlation effect p H1 of 0.40 (Cohen 1988; Lovakov and Agadullina 2021), an α (alpha) of 0.05, and power (1 − β err prob) of 0.8, a sample size of *N* = 46 was obtained. Thus, with a sample of *N* = 54 teachers in the present article, significant moderate effects ought to be detectable with a probability of 80%.

Data were analysed using Mplus 8.1 (Muthen and Muthen 1998–2017) for descriptive and correlational analyses. The full information likelihood (FIML) method was applied to include all available information. The maximum likelihood estimator with standard errors (MLR) was utilised to provide robustness to non-normality. Aggregated data were used to calculate the correlations between teachers’ SRL promotion and students’ SRL skills. For this purpose, class averages were generated and assigned to the respective teacher. Data were analysed using the “type complex” command and class affiliation as cluster variables to account for the nested data.

## Results

### Teachers’ promotion of self-regulated learning

The mean number of directly instructed strategies and teachers time on strategy instruction were computed to assess teachers’ explicit and implicit strategy instruction (see Table 2). Across the analysed videotaped lessons, teachers promoted SRL strategies mainly implicitly (*M* = 17.85 strategies, *SD* = 8.41), and there was hardly any explicit instruction of SRL strategies (*M* = 0.24 strategies, *SD* = 0.80). The time invested by teachers in promoting SRL strategies reflects this finding (implicit: *M* = 5.00 min, *SD* = 2.40, explicit: *M* = 0.09 min, *SD* = 0.38). In addition, teachers’ time invested in promoting SRL strategies was mainly directed towards promoting metacognitive strategies (*M* = 4.19 min, *SD* = 2.17) and secondarily towards cognitive strategies (*M* = 00.35 min, *SD* = 1.38). Teachers did not promote emotional strategies.

**Table 2:** Descriptive Results of Teachers’ Direct Promotion of Self-Regulation

Strategy		Explicit direct promotion			Implicit direct promotion			Total direct promotion		
		*M*	*SD*	Range	*M*	*SD*	Range	*M*	*SD*	Range
*Metacognitive strategies*	Time	00:00	00:00	00:00–00:00	04:19	02:17	00:37–13:16	04:19	02:17	00:37–13:16
	Quantity	00.00	00.00	00.00–00.00	15.74	07.58	05.00–35.00	15.74	07.58	05.00–35.00
Planning and goal setting	Quantity	00.00	00.00	00.00–00.00	12.02	05.25	03.00–32.00	12.02	05.25	03.00–32.00
Monitoring	Quantity	00.00	00.00	00.00–00.00	02.91	04.11	00.00–23.00	02.91	04.11	00.00–23.00
Evaluation and reflection	Quantity	00.00	00.00	00.00–00.00	00.76	01.60	00.00–06.00	00.76	01.60	00.00–06.00
Regulation	Quantity	00.00	00.00	00.00–00.00	00.06	00.23	00.00–01.00	00.06	00.23	00.00–01.00
*Cognitive strategies*	Time	00:08	00:38	00:00–04:19	00:27	01:29	00:00–10:29	00:35	01:38	00:00–10:29
	Quantity	00.19	00.75	00.00–05.00	01.02	01.54	00.00–06.00	01.20	01.77	00.00–07.00
Rehearsal	Quantity	00.00	00.00	00.00–00.00	00.04	00.19	00.00–01.00	00.04	00.19	00.00–01.00
Organization	Quantity	00.00	00.00	00.00–00.00	00.00	00.00	00.00–00.00	00.00	00.00	00.00–00.00
Elaboration	Quantity	00.00	00.00	00.00–00.00	00.30	00.74	00.00–03.00	00.30	00.74	00.00–03.00
Subject specific	Quantity	00.19	00.75	00.00–05.00	00.69	01.10	00.00–05.00	00.87	01.43	00.00–07.00
*Resources strategies*	Time	00:00	00:02	00:00–00:14	00:05	00:10	00:00–00:59	00:05	00:10	00:00–00:59
	Quantity	00.04	00.27	00.00–02.00	00.54	00.77	00.00–03.00	00.57	00.84	00.00–03.00
Attention control/concentration	Quantity	00.04	00.27	00.00–02.00	00.20	00.45	00.00–02.00	00.24	00.58	00.00–03.00
Help seeking	Quantity	00.00	00.00	00.00–00.00	00.11	00.32	00.00–01.00	00.11	00.32	00.00–01.00
Information seeking	Quantity	00.00	00.00	00.00–00.00	00.04	00.19	00.00–01.00	00.04	00.19	00.00–01.00
Regulation of learning environment	Quantity	00.00	00.00	00.00–00.00	00.19	00.55	00.00–00.30	00.19	00.55	00.00–03.00
*Motivational strategies*	Time	00:00	00:01	00:00–00:07	00:05	00:09	00:00–00:38	00:05	00:09	00:00–00:38
	Quantity	00.02	00.14	00.00–01.00	00.56	01.02	00.00–05.00	00.57	01.04	00.00–05.00
Enhancement of control	Quantity	00.02	00.14	00.00–01.00	00.33	00.78	00.00–04.00	00.35	00.78	00.00–04.00
Enhancement of value	Quantity	00.00	00.00	00.00–00.00	00.15	00.53	00.00–03.00	00.15	00.53	00.00–03.00
Enhancement of a growth mindset	Quantity	00.00	00.00	00.00–00.00	00.07	00.26	00.00–01.00	00.07	00.26	00.00–01.00
*Emotional strategies*	Time	00:00	00:00	00:00–00:00	00:00	00:00	00:00–00:00	00:00	00:00	00:00–00:00
	Quantity	00.00	00.00	00.00–00.00	00.00	00.00	00.00–00.00	00.00	00.00	00.00–00.00
*Total*	Time	00:09	00:38	00:00–04:19	05:00	02:40	01:09–15:07	05:10	02:42	01:38–16:36
	Quantity	00.24	00.80	00.00–05.00	17.85	08.41	06.00–37.00	18.09	08.34	06.00–37.00

Individual case high scores for ‘Total direct promotion’ may lead to a higher range than in the separated ranges for ‘Explicit direct promotion’ and ‘Implicit direct promotion’; As the mean values are rounded, there may be deviations between the summed mean values for ‘Explicit direct promotion’ and ‘Implicit direct promotion’ compared to the mean value for ‘Total direct promotion’

*M* mean, *SD* standard deviation

Teachers’ indirect SRL promotion was computed using the mean scores on each of the six constructs (see Table 3) for designing powerful SRL learning environments (overview constructs and example items, see Appendix Table 6). Teachers primarily designed SRL learning environments that support students’ positive emotions (student support; *M* = 3.15, *SD* = 0.64). In contrast, teachers created learning environments which the least supportive conditions for fostering students’ self-determined learning (*M* = 1.72, *SD* = 0.65).

**Table 3:** Descriptive Results of Teachers’ Indirect Promotion of Self-Regulated Learning

Construct	*M*	*SD*	Skew	Kurtosis	Observed range	Possible range
Self-determination	1.72	0.65	1.56	2.51	1.00–4.00	1.00–4.00
Value	2.41	0.71	0.28	−0.71	1.33–4.00	1.00–4.00
Success expectation	2.57	0.51	0.25	−0.68	1.50–3.75	1.00–4.00
Cooperative learning	2.65	0.95	−0.34	−0.93	1.00–4.00	1.00–4.00
Student support	3.15	0.64	−0.67	−0.29	1.50–4.00	1.00–4.00
Constructivist learning	2.64	0.70	−0.03	−0.30	1.00–4.00	1.00–4.00

1 = low, 4 = high

*M* mean, *SD* standard deviation

### Relation between teachers’ professional competences and their promotion of self-regulated learning

Analysing the relationship between teachers’ professional competence in SRL and their SRL promotion yields only a few significant correlations (see Table 4). Concerning direct promotion, significant positive correlations are found for motivational regulation skills and direct promotion of metacognitive strategies (*r* = 0.36, *p* < 0.001), as well as for the CK-SRL meta and direct promotion of resource strategies (*r* = 0.33, *p* = 0.012). Further, negative correlations are found concerning the direct promotion of metacognitive strategies and metacognitive regulation skills (*r* = −0.30, *p* = 0.008) and the malleability mindsets about SRL (*r* = −0.24, *p* = 0.017). The direct SRL promotion also negatively correlates with teachers’ CK-SRL (*r* = −0.22, *p* = 0.030).

**Table 4:** Correlations Between Teachers’ Professional Competence in Self-Regulated Learning and Their Promotion of Self-Regulated Learning

Construct	Teachers’ direct SRL promotion				Teachers’ indirect SRL promotion					
	Metacognitive strategies	Cognitive strategies	Resources strategies	Motivational strategies	Self-determination	Value	Success expectation	Cooperative learning	Student support	Constructivist learning
Metacognitive awareness	−0.11	0.07	0.18	0.02	0.01	−0.01	−0.36**	−0.02	0.34*	−0.10
Metacognitive regulation skills	−0.30**	−0.00	−0.20*	0.07	0.06	0.06	0.02	0.20	−0.41**	0.16
Motivational regulation skills	0.36**	0.09	−0.04	−0.21	−0.33**	−0.08	0.35**	−0.07	−0.09	−0.00
CK-SRL	0.20	−0.22*	−0.03	0.19	0.05	−0.01	−0.02	0.17	−0.03	0.12
CK-SRL Meta	−0.05	0.19	0.33*	0.02	−0.15	0.02	0.00	0.14	0.18	−0.13
PCK-SRL	0.01	−0.17	0.09	−0.09	0.10	−0.36**	−0.12	0.32**	0.08	0.48**
Malleable mindsets	−0.24*	0.16	0.04	0.07	0.01	0.16	−0.14	0.01	−0.22*	−0.24*
Relevance mindsets	0.11	−0.07	−0.20	0.13	−0.00	0.08	−0.07	−0.05	0.11	0.02

^*^p < 0.05, ^**^p < 0.01 (two-tailed); **bold**: significant values after Bonferroni correction of the significance level: α_*adj*_ = 0.05/80 = 0.000625

Regarding the indirect promotion (see Table 4), significant positive correlations can be found for the promotion of success expectation and teachers’ motivational regulation skills (*r* = 0.35, *p* < 0.001), the creation of cooperative learning environments and teachers’ PCK-SRL (*r* = 0.32, *p* = 0.002), student support and teachers’ metacognitive awareness (*r* = 0.34, *p* = 0.014), and teachers’ creation of a constructivist learning environment and their PCK-SRL (*r* = 0.48, *p* < 0.001). Negative correlations can be found in granting self-determination and teachers’ motivational regulation skills (*r* = −0.33, *p* = 0.001), the value promotion and teachers’ PCK-SRL (*r* = −0.36, *p* = 0.003), the fostering of success expectation and teachers’ metacognitive awareness (*r* = −0.36, *p* = 0.004), student support and teachers’ metacognitive regulation skills (*r* = −0.41, *p* < 0.001) as well as malleability mindsets about SRL (*r* = −0.33, *p* = 0.030), and the opportunity for constructivist learning and teachers’ malleability mindsets about SRL (*r* = −0.24, *p* = 0.019). All other constructs investigated show no significant correlations (see Table 4).

After Bonferroni correction of the significance level for multiple testing to avoid alpha error accumulation, only the correlations concerning direct promotion of metacognitive strategies and teachers’ motivational regulation skills, and concerning the indirect promotion of success expectation and teachers’ motivational regulation skills, between teachers’ student support and their metacognitive regulation skills, and for teachers’ creation of a constructivist learning environment and teachers’ PCK-SRL proved to be statistically significant. However, since the Bonferroni correction is very conservative and can also increase the type 2 error, the uncorrected significances are also reported (see Table 4).

### Relation between teachers’ promotion of self-regulated learning and their students’ self-regulated learning

The analyses mainly revealed insignificant correlations between teachers’ SRL promotion and their students’ SRL (see Table 5). Only two significant correlations were found regarding teachers’ direct SRL promotion: While the metacognitive strategies promoted were negatively related to students’ cognitive regulation skills (*r* = −0.44, *p* < 0.001), the cognitive strategies promoted were positively correlated with students’ cognitive regulation skills (*r* = 0.28, *p* = 0.024). Furthermore, teachers’ indirect SRL promotion concerning self-determination is positively related to students’ motivational regulation skills (*r* = 0.28, *p* = 0.031) and negatively to students’ use of strategies (*r* = −0.28, *p* = 0.011). Moreover, promoting the success expectation correlates positively with the learners’ use of strategies (*r* = 0.55, *p* < 0.001). Also, promoting constructivist learning is positively related to the learners’ motivational regulation skills (*r* = 0.29, *p* = 0.026). All other constructs examined show no significant relationships (see Table 5).

**Table 5:** Correlations Between Students’ Aggregated Skills in Self-Regulated Learning and Teachers’ Promotion of Self-Regulated Learning

Construct	Teachers’ direct SRL promotion				Teachers’ indirect SRL promotion					
	Metacognitive strategies	Cognitive strategies	Resources strategies	Motivational strategies	Self-determination	Value	Success expectation	Cooperative learning	Student support	Constructivist learning
*Students’ aggregated Skills in Self-Regulated Learning*										
Metacognitive knowledge	−0.20	−0.03	0.00	0.20	−0.08	−0.20	−0.03	−0.14	0.03	0.22
Cognitive regulation skills	−0.44**	0.28*	0.02	−0.07	0.12	0.18	0.09	0.11	−0.21	−0.20
Motivational regulation skills	0.08	−0.23	−0.05	0.16	0.28*	−0.12	−0.24	0.20	0.15	0.29*
Strategy use	0.11	−0.03	0.02	−0.14	−0.28*	−0.02	0.55**	−0.23	−0.08	−0.01

^*^p < 0.05, ^**^p < 0.01 (two-tailed); **bold**: significant values after Bonferroni correction of the significance level: α_*adj*_ = 0.05/40 = 0.00125

After Bonferroni correction of the significance level for multiple testing to avoid alpha error accumulation, only the correlations concerning teachers’ direct promotion of metacognitive strategies and students’ cognitive regulation skills and concerning teachers’ indirect promotion of the success expectation and students’ use of strategies proved to be statistically significant. However, since the Bonferroni correction is very conservative and can also increase the type 2 error, the uncorrected significances are also reported (see Table 5).

## Discussion

This study aimed to explore the potential relationship between teachers’ professional competences in SRL and their SRL promotion, as well as to examine whether teachers’ SRL promotion is correlated with students’ SRL skills. The present study focused on observing teachers’ SRL promoting to go beyond self-reported SRL promotion. In the following sections the main findings and implications for future research are discussed.

### Teachers’ observed promotion of self-regulated learning

The first research question addressed the extent to which teachers promote SRL in classes. Analysis of the videotaped lessons reveals that teachers mainly promote strategies implicitly, confirming hypothesis H1a and aligning with previous video studies (e.g., Dignath and Büttner 2018; Kistner et al. 2010). It’s possible that teachers believe implicit strategy instruction is enough to provide students with the necessary information about SRL (Kistner et al. 2010). However, it is also conceivable that explicit strategy instruction occurred beforehand, and teachers now merely recall strategies implicitly. Depending on students’ SRL expertise, teachers might first teach strategies explicitly and then move into implicit and indirect support (Karlen et al. 2020). Longitudinal studies are necessary to analyse changes in teachers’ SRL promotion from explicit to implicit and direct to indirect over time. Research should investigate how well teachers adapt their SRL instruction to students’ needs and how this might impact students’ SRL development.

Regarding the different promoted strategies, the results reveal that teachers spent more time promoting metacognitive rather than cognitive strategies, differing from our expectation (H1b) and previous study results (e.g., Dignath and Büttner 2018). In [country], teachers are encouraged to communicate the lesson plan and goal(s) at the beginning of a lesson. Both were coded as implicit instructions to support students’ metacognition. In doing so, teachers intuitively act as metacognitive models (e.g., Paris and Paris 2001). Another reason for the discrepancy from other studies could be the emphasis on different teaching subjects. We included teachers from various subjects, while Kistner et al. (2010) and Dignath and Büttner (2018) focused on math teachers. Strategy facilitation may be easier or more challenging for teachers depending on the subject and learning objectives (Dignath and Veenman 2021). The extent to which teachers’ SRL promotion is subject-dependent remains an open question (e.g., Greene et al. 2015).

Concerning teachers’ indirect SRL promotion, the observed lessons showed higher mean values in the SRL dimensions of student support, followed by cooperative learning, constructive-oriented learning, and support of success expectation. Lower mean values were found for highlighting the value of the learning content and granting self-determination. Teachers often exhibit indirect SRL promotion, but transferring more responsibility to students is the greatest improvement. Ideally, teachers increasingly delegate the responsibility of structuring and self-regulating learning to students. SRL varies along a continuum from external- to self-regulation, depending on students’ developmental stage (Karlen et al. 2022). Teachers face the challenge of designing adaptive learning environments for students at different SRL stages.

### Teachers’ professional competences and promotion of self-regulated learning

The second research question explored the relationship between teachers’ professional competences of SRL and their SRL promotion. The analysis reveals hardly any connections, thus rejecting our second hypothesis. The literature rarely illustrates significant relationships between teachers’ professional competencies in SRL and their SRL promotion as assessed with video data (e.g., Dignath and Büttner 2018; Spruce and Bol 2015), in contrast to studies using self-reported data of teachers SRL promotion (e.g., Barr and Askell-Williams 2019; Dignath-van Ewijk 2016; Karlen et al. 2023). Moreover, Spruce and Bol (2015) found inconsistencies between teachers’ self-reported data and observed SRL promotion. Multiple perspectives, including teacher-reported data, observations, and students’ perceptions, could be crucial for a comprehensive picture of the relationship between teachers’ professional competences in SRL and their SRL promotion (Karlen et al. 2023). Previous research reveals that other aspects, such as motivation, are also crucial in teachers’ SRL promotion (e.g., Dignath-van Ewijk 2016). Teachers’ expectations of successfully promoting SRL and the value they ascribe to SRL promotion are significant predictors of their self-reported SRL promotion (Jud et al. 2023). Future studies should consider how and which professional competences to assess, as well as how to integrate different perspectives on SRL promotion.

### Relation between teachers’ promotion of self-regulated learning and students’ self-regulated learning

The third research question concerns the relationship between teachers’ SRL promotion and students’ SRL. The analysis yields no clear correlational pattern between teachers’ promotion and students’ SRL skills, as hardly any significant correlations were found. This result is partly aligned with previous results, which found positive (e.g., Depaepe et al. 2010; Moely et al. 1992), no or even negative relationships concerning teachers’ SRL promotion and students’ SRL skills (e.g., Hamman et al. 2000; Heirweg et al. 2021; Karlen 2016). A possibility for the lack of correlations could be due to cross-sectional data, which only provide a short-term perspective on teachers’ SRL promotion. They do not allow conclusions how teachers’ SRL promotion is students’ prerequisites oriented and adaptive in a long-term process with direct and indirect instructions (Dignath and Veenman 2021; Karlen et al. 2020). Furthermore, individual differences among teachers (e.g., individual perceptions about the challenges of promoting SRL) and students (e.g., personal readiness and willingness to engage and develop SRL skills) are likely to contribute to the inconsistent link between SRL promotion and student SRL skills. These individual differences can impact the nature and strength of correlations between teachers’ SRL promotion and students’ SRL skills. We used a multilevel approach to detect differences at the teacher/class level (teacher competence and students aggregated SRL skills). In future studies, it could be valuable to explore effects at the student level by combining both analytical levels (teacher/class level and students’ level). Additionally, it might be important to consider and include additional factors at the school level.

### Limitations and future studies

The study has several limitations to consider when interpreting the findings.

Only one lesson was observed per teacher. The results should be interpreted and compared cautiously, as it remains unclear how many lessons are needed to capture the quality of teachers’ instructions (Praetorius et al. 2014). Video recordings across several lessons could provide more insight, but resource-intensive analysis must be set concerning the additional information gained.

Unlike other video-based classroom studies, teachers in our sample taught different subjects, which might impact SRL promotion. Comparisons to other studies should be cautious (Dignath and Veenman 2021). Examining teachers’ SRL promotion across different subject areas can offer valuable insights into subject-specific variations in SRL promotion. These differences can impact the professional development of teachers concerning SRL and their subjects.

Low variance in teachers’ observed SRL promotion may affect detecting relationships between teachers’ professional competences, their SRL promotion, and their students’ SRL skills. Aggregating students’ SRL outcomes at the class level could reduce variance, while SRL might vary more significantly at the individual student level. Both teachers and students exhibit significant individual differences, which can impact the strength and direction of correlations. Future studies examining these differences, for example, through cluster analysis, could uncover links between teachers’ SRL promotion and students’ SRL. Such studies could provide valuable insights into the extent to which teachers’ SRL support varies and how students benefit from SRL promotion with different abilities and conditions (e.g., Moely et al. 1992; Zepeda et al. 2019).

More sensitive instruments to assess teachers’ and students’ SRL may be needed to capture the complexity of these constructs. The cross-sectional design limits the ability to establish causal relationships. Longitudinal analysis may reveal dynamics in teachers’ adaptive SRL promotion and students’ SRL development. Larger samples, multiple lessons per teacher, and longitudinal designs would enhance our understanding of these complex dynamics.

### Practical implications

The results on teachers’ SRL promotion emphasise the need for improvement in explicitly teaching SRL strategies. Additionally, emotional strategies were not incorporated into teachers’ instruction. Teachers might benefit from professional development programs and training sessions focusing on enhancing their knowledge and skills related to the direct SRL promotion. Such professional development can include workshops on effective instructional strategies, integrating emotional regulation strategies, and fostering student autonomy and responsibility in their learning. Encouraging collaboration among teachers within and across subject areas can facilitate the sharing of best practices in promoting SRL (Perry et al. 2020). Teachers can exchange strategies, resources, and experiences to enhance their students’ SRL support.

### Conclusion

The study revealed that the participating teachers demonstrated limited direct SRL promotion, primarily fostering strategies implicitly while dedicating most of their time to promoting metacognitive strategies. Their designed learning environments primarily emphasized student support and facilitated cooperative learning. Other central aspects of indirect SRL promotion, such as granting self-determination, were less frequently observed. The analyses lacked significant correlations between teachers’ SRL competences and their SRL promotion, as well as between teachers’ SRL promotion and students’ SRL. Future studies may benefit from employing a combination of multiple measures to assess teachers’ SRL promotion. By using a comprehensive approach that incorporates various measurement tools, such as direct observation over a more extended period, self-report questionnaires, and student assessments, researchers can gain a more holistic understanding of teachers’ SRL promotion. Finally, further investigations should examine the duration and intensity of promoting SRL to determine the timeframe and level of intensity of SRL promotion required for effects to manifest in students.

## Appendix

​

**Table 6:** Constructs and Sample Items for the High-Inference Coding of Teachers’ Indirect Promotion

Construct (Number of items)	Sample Item
Self-determination (4)	The teacher allows the students to take responsibility for structuring their learning by giving them some decision-making freedom.^*^
Value (3)	Learning is integrated with a real-life context.^*^
Success expectation (4)	The teacher allows the students to choose between different tasks (e.g., alternatives and/or difficulty levels).^**^
Cooperative learning (3)	The teacher ensures that the students work together cooperatively and intervenes if necessary.^*^
Student support (4)	The teacher shows the students that they take them seriously by asking positive questions (e.g., “Aha, and how did you do that exactly?”).^**^
Constructivist learning (3)	The teacher integrates new knowledge in a meaningful context and/or introduces new knowledge by creating a cognitive conflict.^*^

We extended the ATES with two constructs: success expectation and student support

^*^Dignath et al. (2022)

^**^Hugener et al. (2006)
